# Supplementary material for: Association between oral microbiome and seven types of cancers in East Asian population: a two-sample Mendelian randomization analysis
Source: Front Mol Biosci. 2023 Nov 21;10:1327893. doi: 10.3389/fmolb.2023.1327893 (PMC10702768; doi:10.3389/fmolb.2023.1327893)
Supplement: Supplementary file 1 [file DataSheet1.ZIP › Supplementary Materials/MR plots for tongue/tongue═╝/Pancreatic cancer/╥╚╧┘░⌐_╔╕╤í╜ß╣√/pheno.3794_to_pancreatic cancer_forest.pdf]

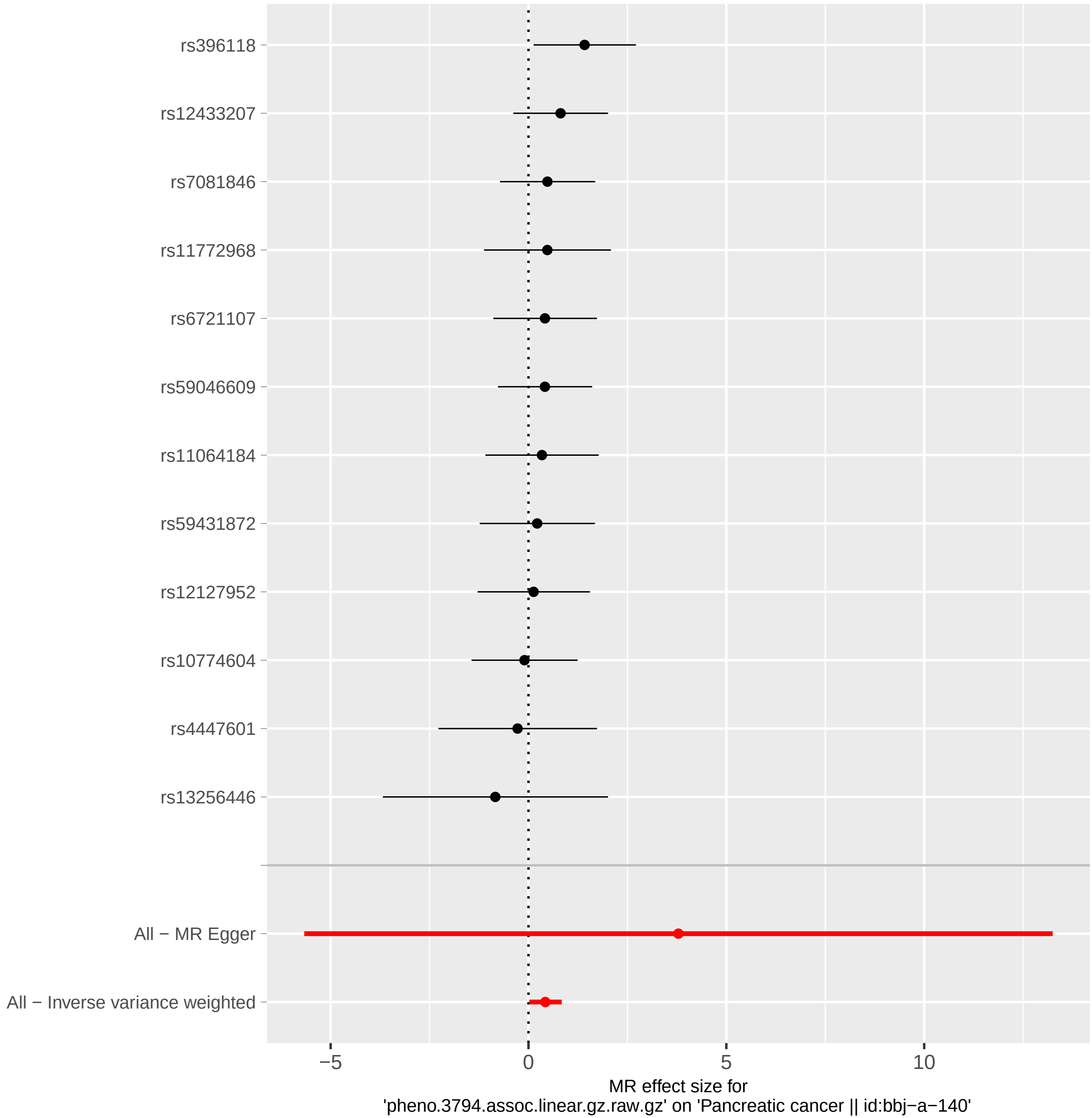
rs396118
rs12433207
rs7081846
rs11772968
rs6721107
rs59046609
rs11064184
rs59431872
rs12127952
rs10774604
rs4447601
rs13256446
All − MR Egger
All − Inverse variance weighted
−5
0
5
10
MR effect size for
'pheno.3794.assoc.linear.gz.raw.gz' on 'Pancreatic cancer || id:bbj−a−140'
